# Has Cognitive Ability Become More Important for Education and the Labor Market? A Comparison of the Project Talent and 1979 National Longitudinal Survey of Youth Cohorts

**DOI:** 10.3390/jintelligence11080169

**Published:** 2023-08-21

**Authors:** Gary Neil Marks

**Affiliations:** Department of Sociology, Social and Political Sciences, University of Melbourne, Melbourne 3010, Australia; gmarks@unimelb.edu.au

**Keywords:** grades at school, educational attainment, occupational attainment, income

## Abstract

Modernization and meritocratic theories contend that with modernization, socioeconomic background (SES) becomes less important for educational and socioeconomic attainments, while cognitive ability becomes more important. However, the evidence is mixed. This study investigates if the effects of SES and cognitive ability on educational and labor market outcomes have changed in the US by comparing two longitudinal cohort studies: the 1960 Project Talent and the 1979 National Longitudinal Survey of Youth. For all outcomes—grades-at-school, educational and occupational attainment, and income—cognitive ability clearly has stronger effects than a composite and broad measure of SES. The effects of cognitive ability for grades-at-school and income are notably stronger in the more recent cohort, whereas its effects on educational and occupational attainment are similar. SES effects, net of ability, for educational and occupational attainment are only moderate and for school grades and income are very small (β < 0.10). However, for each outcome SES effects are stronger in the more recent NLSY79 cohort. This is attributed to ability being a stronger influence on the educational and socioeconomic attainments of NLSY79 parents compared to Project Talent parents. These analyses suggest that in the US, cognitive ability has long been an important, and SES a much weaker, influence on educational and subsequent socioeconomic outcomes.

## 1. Introduction

According to modernization theory, cognitive ability is increasingly important as modernization occurs. According to Levy (1966, p. 218), modern societies require ‘experts’ that rationally apply scientific knowledge to solve problems. Modernization is characterized by “rationality, universalism, and functional specificity” (Levy 1966, p. 240). In their landmark book *The American Occupational Structure*, Blau and Duncan (1967, p. 429) conclude that American society is characterized by ‘expanding universalism’ as universalistic criteria replace particularistic criteria (e.g., social class, ethnicity, gender). Status is no longer directly inherited but must be legitimated by actual achievements that are socially acknowledged, most commonly through educational qualifications. In an achievement orientated society, education is the primary mechanism for socioeconomic attainments (Blau and Duncan 1967, p. 430). A universalistic orientation means that “differential awards are distributed on the basis of differences in abilities, whether native or acquired” (Blau and Duncan 1967, p. 440).

The importance of education for occupational attainment and income is well established. However, its importance is not always interpreted as increased universalism. Instead, the more widely accepted interpretation is that education reinforces, or even strengthens, family-of-origin socioeconomic inequalities (Collins 1971; Bourdieu 1977; Goldthorpe 1996). Therefore, sociologists and other social scientists tend to reject modernization theory (Goldthorpe 1996; Hout and DiPrete 2006, p. 8).

Nielsen (2008, p. 17) argued that increasing genetic effects, often associated with cognitive ability, are responsible the apparent failure of modernization theory, that is the absence of unequivocal reductions in the intergenerational reproduction of socioeconomic inequalities.

The related meritocratic thesis is that merit, typically defined as ability plus effort, becomes the most important factor for educational, occupational, and economic attainments (Bell 1973, p. 44; Herrnstein 1973). Herrnstein (1973, p. 61) defined meritocracy as “the advancement of people on the basis of ability, either potential or fulfilled, measured objectively”. Unlike other economic systems, meritocracies are characterized by both economic efficiency and fairness (Wooldridge 2021, p. 367). Theoretically, because of their economic efficiency, meritocracies generate large enough surpluses to provide safety nets and other types of social welfare.

### 1.1. Empirical Evidence

The importance of cognitive ability^1^ for social stratification has a long history. Over a century ago, Burt (1917, p. 52) estimated correlations between 0.4 and 0.6 for general intelligence with primary school students’ performance in specific subjects. For the 1921 graduating class at Grover Cleveland High School St Louis, IQ correlated at around 0.5 with average achievement and at 0.7 with performance in English (Shewman 1926, p. 138). Benson (1942), and later Bajema (1966, p. 307), reported correlations of around 0.6 between cognitive ability and educational attainment. Burt (1943) argued that income inequality is largely, though not entirely, an indirect result of inequalities in cognitive ability. Anderson et al. (1952) estimated that at least one-third of father-to-son occupational mobility can be attributed to intelligence. According to Lipset and Bendix (1959, p. 236) “intelligence is a major dynamic factor affecting mobility in all societies in which educational achievement or other qualities associated with intelligence play an important role in status placement”. Duncan (1968, p. 11) contends that ability “serves as a kind of springboard, launching many men into achievements removing them considerable distances from the social class of their birth”. This hypothesis was confirmed by Waller (1971); the amount and direction of social mobility was correlated with discrepancies between sons’ cognitive abilities and their social class of origin. Strenze’s (2007, p. 412) meta-study of studies published between 1929 and 2003 that found strong correlations of childhood or adolescent ability with educational attainment (r ≈ 0.55) and occupational attainment (r ≈ 0.45) and much weaker correlations with income (r ≈ 0.20), with no systematic change over time. These studies show that cognitive ability has been important for socioeconomic attainments for a long time.

### 1.2. Changes over Time

An important implication of modernization and meritocratic theories is that the impact of cognitive ability has increased over time. However, there is no clear evidence that the effects of ability on socioeconomic attainments have increased over time, at least since World War II.

Comparing the 1979 and 1997 National Longitudinal Survey of Youth studies, Marks (2022, pp. 7, 8) found that the correlation between latent general cognitive isolated from a battery of cognitive tests (*g*) and high school grades was weaker in the more recent NLSY97 cohort (0.44 cf. 0.60).

Comparing IQ scores of U. S. high school graduates that did and did not enter college, Taubman and Wales (1972, pp. 17–24, 19) found “a very pronounced upward trend” in the role of ability between 1921 and the early 1960s. In the 1920s, about 60% of the most able students (the top ability decile) went to college, while in the 1960s the comparable figure was over 90%. Herrnstein and Murray (1994, p. 35) updated this analysis for the 1980s and found no change between the 1960s and 1980s. Contrary to modernization theory, Jencks et al. (1979, p. 86) report that the correlation of test scores with educational attainment has remained stable since World War I, at around 0.55. There is some indication that correlations have increased slightly since the 1970s, with recent studies reporting correlations closer to 0.6 and higher (Zagorsky 2007, p. 493; Zisman and Ganzach 2021, Tables A1 and A2; Marks 2022, p. 7). Strenze (2007, p. 413) reported a small decline in the IQ-education correlation by year of success (1929 to 2003), so there was no upward historical trend. Net of parents’ education and family income, the standardized coefficient for ability on educational attainment was the same (β ≈ 0.45) in the 1979 and 1997 NLSY studies (Marks 2022, p. 7).

The correlations between ability and occupational attainment are also remarkably stable. Comparing cohorts from two studies, Jencks et al. (1979, p. 101) concluded that there is little support for the hypothesis that occupational attainment has become more dependent on skills measured by standardized tests. They even suggest that test scores may have had stronger effects on initial occupation among cohorts born *before* World War II. Strenze (2007, p. 413) reported no discernable trend in the IQ-occupational attainment correlation by year of success. Marks (2022) reported a weaker correlation between general cognitive ability (*g*) and average occupational attainment (for ages 25 to 39) in the more recent NLSY97 cohort (r = 0.46) compared to the 1979 cohort (r = 0.54). Net of parents’ education and income, and educational attainment, the impact of cognitive ability on occupational attainment was slightly weaker in the NLSY97 than in the NLSY79 (β ≈ 0.18 cf. β ≈ 0.22).

The evidence for increasing effects of ability on income is also equivocal. Jencks et al. (1979, p. 101) report no change in the IQ-income correlation by age group in Kalamazoo study men born in 1916 or 1917. A comparison of the earnings of 24-year-olds in two American Longitudinal studies, the Class of 1972 (NLS72) and High School and Beyond (HS&B), found larger effects for basic cognitive skills, net of family background and education and labor force experience in the 1982 HS&B study than in the 1972 study (Murnane et al. 1995, pp. 258–59). However, in a later publication, Murnane et al. (2000, pp. 556–57) found that, for men, the percentage effect was larger in the older NLS72 study than in the HS&B. Grogger and Eide (1995, pp. 292–94) found that the increase in the wage premium for college could partially be attributed to increases in the returns to ability. In 1978, a one standard deviation increase in mathematics ability was associated with a 2% increase in wages. By 1987 this effect had grown to 5% for men and 7.5% for women (Grogger and Eide 1995, p. 292). Strenze (2007, p. 413) reported fluctuating correlations between IQ and income. Marks (2022, p. 7) reported a slightly stronger correlation between *g* and average logged income in the NLSY97 (r = 0.41) than in the NLSY79 (r = 0.38). Net of parents’ education and income, and educational and occupational attainment, the impact of a one-standard deviation increase ability on income was stronger in the NLSY97 (a 21% increase) than the NLSY79 (a 11% increase).

### 1.3. Present Study

Evidence for modernization and meritocrat theses is mixed. Some studies find stronger effects for cognitive ability, whereas most others find no change. The purpose of this study is to assess the contentions of modernization and meritocratic theories by comparing the effects of ability vis-à-vis socioeconomic background on stratification outcomes by analysis of two US longitudinal cohort studies. The stratification outcomes are grades-at-school, educational attainment, occupational attainment, and (logged) income at age 29.

## 2. Materials and Methods

### 2.1. Data

The data analyzed is from Project Talent and the NLSY79 longitudinal cohort studies. 

Project Talent is a national longitudinal study developed by the American Institutes for Research (see Flanagan 1960/1976; Wise et al. 1979). The Project Talent data was derived from a random probability sample of students attending secondary schools (public, parochial, and private). The sample comprised a total of about 440,000 grade 9 to 12 students from more than 1350 secondary schools. Ninety-seven percent of the students were born between 1941 and 1945. Students completed a comprehensive two-day battery of tests of aptitudes, abilities, achievements, interests, and background factors (Orr 1961; Shaycoft 1977, p. 2; Wise et al. 1979, p. 10).

The Project Talent follow-up surveys were conducted 1, 5, and 11 years after high school graduation to investigate the respondents’ educational and labor market careers and personal situations. The data for these analyses are from the 11-year follow-up, conducted between 1971 and 1974 when respondents were 29 years old (Wise et al. 1979, p. 2). The response rate to the 11-year follow-up was 22.6% (Wise et al. 1979, p. 16).

The NLSY79 is a household probability sample of 12,686 adolescents aged between 14 to 21 on 31 December 1978, that is born between 1957 and 1964. As such, they are 12 to 23 years younger than the Project Talent cohort. The NLSY79 comprises 6111 respondents from the original household probability sample, 5295 respondents from the minority and ‘poor white’ oversample, and 1280 military sample respondents. Respondents were interviewed annually from 1979 to 1994 and biennially since 1994 (Cooksey 2018; BLS 2022a). For comparability with the 11-year follow-up Project Talent data, the measures of the NLSY79 outcome variables were constructed from data collected when respondents were 29 years old.

Since there were no follow-up studies for Project Talent between 11 and 50 years after graduating high school, it is not possible to compare the two cohorts during prime working age. The analyses could not include the NLSY97 since the NLSY97 did not collect data on father’s and mother’s occupations. Occupation, especially father’s occupation, is a central variable in stratification research.

### 2.2. Measures

#### 2.2.1. Cognitive Ability

For the computation of *g* from the Project Talent data, responses to 15 subtests were analyzed by confirmatory factor analysis of a single latent factor. Wise et al. (1979, p. 22, Appendix A) detail the subtests’ content, numbers of items, reliabilities, and intercorrelations. Table 1 presents the loadings on the latent factor of the 15 Project Talent subtests. The loadings on the latent factor are highest for reading comprehension (0.85), vocabulary I (0.85), math information (0.80), and English literature (0.79), and are lowest for visualization in two dimensions (0.46), advanced high school math (0.55), and arithmetic computation (0.56). The latent measure of cognitive ability (*g*) used in this study is similar to that isolated by Jencks et al. (1983, p. 61) from 30 Project Talent subtests since the loadings are most often very similar (Table 1, last column).

For the NLSY79, 12 subtests were analyzed by confirmatory factor analysis of a single latent factor. The loadings were reported by Marks (2022, p. 5).

For both data sets, the *g* measures computed from the loadings and respondents’ scores on the subtests. They were standardized and adjusted for age by taking the residuals from regressions on the age students took the tests.

The Project Talent and NLSY79 *g* measures are comparable. *g* factors isolated from different intelligence tests correlate very highly, often above 0.95 (Jensen 1998, pp. 81–83; Johnson et al. 2004, 2008; Floyd et al. 2013).

#### 2.2.2. High School Grades

For Project Talent, students were asked their grades in English, foreign languages, social studies, mathematics, and science from grade 9 to the middle of grade 11. The responses were averaged into a single variable (BY_P820). Nathan, Credé, and Thomas’s meta-study (Kuncel et al. 2005, p. 72) reported a correlation of 0.82 between self-reported grades and actual grades. However, the reliability of grades is associated with student performance (reports from lower achieving students are less reliable) and, to a lesser extent, with cognitive ability. They advise caution in interpreting the findings from analyses of self-reported grades.

For the NLSY79, grades were obtained from the 1979 High School Transcript Survey. Grades were recorded for a total of 64 subjects for grades 9 to 12, scored on a five-point scale ranging from 0 for an E, F, or fail, to 4 for an A (BLS 2022b). The small proportions coded 6 (pass) were declared missing.

For these analyses, average high school grade was simply the average of all grades recorded without regard to grade level, subject difficulty, calendar year, or high school attended. The measure ranged from 0 to 50 in Project Talent and 0 to 4 in the NLSY79.

#### 2.2.3. Educational Attainment

Years of education was constructed from the “Amount of Education Completed” variable in the 11-year follow-up (Y11_E001) data (Wise et al. 1979, p. 40). Scores were converted to formal years of education; for example, a score of 8 for completing grade 8, 9 for completing grade 9, 12 for high school graduation (without post-school education), 16 for a completed bachelor’s degree, 18 for a master’s degree, and 20 for a doctorate, medical, or law degree.

Educational attainment in the NLSY79 is measured by the highest grade completed, equivalent to the number of years of formal education. The measure ranges from zero to 20 years of formal education. It is comparable to the Project Talent educational attainment measure.

#### 2.2.4. Occupational Attainment

In the 11-Year Project Talent follow-up, occupations were coded according to a 4-digit Occupational Coding Schema detailed in an appendix of the Project Talent codebook (Flanagan 1960/1976). The occupation codes were converted to the 1970s census occupational codes and then converted to socioeconomic (SEI) scores^2^ according to the detailed mapping of Featherman et al. (1975).

For the NLSY79, respondents’ occupations were coded according to the 1980 census occupational classification for NLSY79 survey waves conducted between 1984 and 2000. The 1980 occupational codes were recoded to Socioeconomic Index (SEI) scores using correspondence tables (Featherman et al. 1975; Nakao and Treas 1994). For the 2000 classification, the codes are first converted to the 2010 occupational schema (there are only minor differences between the 2000 and 2010 schemas) and converted to SEI scores according to the correspondences detailed by Hout et al. (2014).

#### 2.2.5. Income

For the 11-year Project Talent follow-up, respondents were asked their total personal earnings from all sources for the previous year (Y11_O213)^3^. For the NLSY79, personal income was measured by the sum of employment and farm or business income, for comparability with Project Talent. Incomes from both studies were logged using the inverse hyperbolic sine transformation (IHS), which reduces positive skew (Friedline et al. 2015)^4^. Respondents with zero income were not included in the analyses of income.

#### 2.2.6. Socioeconomic Background (SES)

The composite measure of socioeconomic background (SES) is based on five SES indicators: father’s and mother’s education and occupational attainment, and family income. Family wealth was not included since it was poorly measured in Project Talent and, in both datasets, has lower correlations with SES than the other SES indicators.

For Project Talent, father’s and mother’s education was elicited by a question asking students’ the highest level of education achieved by each parent (Wise et al. 1979, p. 38). As for respondents’ education, parents’ education was recoded into approximate years of formal education, ranging from 4 (none, or some grade school) to 20 (completed a PhD or a post-graduate professional degree).

Students were asked two questions on parents’ occupations. The first asked students to choose their parents’ occupation from 17 occupational groups, ranging from unskilled to professional work. A second question asked what profession or technical work. Students were asked to choose an occupation that came closest to their parent’s occupation from a list of 33 occupations (see Flanagan 1972, pp. 18–19). From these two questions, measures of SEI scores were constructed for both parents utilizing the mapping of Hout et al. (2014).

Family income was constructed from student responses to family income (BY_SIB173). Students could choose between 5 income categories with a “I can’t estimate this” option, which was selected by nearly 40% of students. Family income was measured by the midpoints of each category. For the last category ($12,000 or more), the Pareto estimate ($19,662) was used (see Parker and Fenwick 1983).

The NLSY79 collected data on the highest grade ever completed for respondents’ fathers and mother’s education. Father’s and mother’s occupational SEI scores were constructed from 1970 occupational codes. SEI scores were constructed in the same way as respondents’ occupations. Total net family income for the previous year was obtained from interviews with parents in 1979 and IHS transformed.

The SES measures were constructed by first standardizing each variable and then averaging the non-missing values. This SES measure correlates at 0.65 with the already constructed Project Talent SES measure, which included a much larger number of variables, including books in the home and consumables.^5^

Since 40% of responses to the Project Talent family income question are missing and the question was answered by students rather than their parents, the Project Talent SES measure is less reliable than the NLSY79 measure. However, the correlations between family income and SES are comparable: 0.61 in Project Talent and 0.60 in the NLSY79 (see Table 2). In both studies, the dominant components of the SES measures are father’s and mother’s education and occupation.

### 2.3. Statistical Methods

#### 2.3.1. Models

For each outcome, a series of models were estimated. The first model comprises SES only. Model 2 comprises only cognitive ability (*g*). Model 3 comprises both SES and cognitive ability. For occupational attainment and income, model 4 adds educational attainment. For logged income, model 5 adds occupational attainment. Across the two cohorts, the models are identical and the measures, as far as possible, the same. To facilitate comparisons of the relative importance of SES and cognitive ability, the standardized coefficients are included in the tables (italicized) and referred to in the results section.

Proc Calis in SAS was used to obtain Maximum Likelihood estimates (Madhanagopal and Amrhein 2019). The estimates are equivalent to the estimates obtained from Ordinary Least Squares multiple regression.

#### 2.3.2. Weighting

For Project Talent, the analyses were weighted by the appropriate weight (y11_wgta) for the 11-year follow-up (Wise et al. 1979, pp. 47–53). For the NLSY79, weights were constructed from the available sample and attrition weights for respondents when they were 29 years old.

#### 2.3.3. Missing Data

Since the samples are large and there is little missing data for SES and cognitive ability, missing data was handled list-wise. Initial analyses employed Full Information Maximum Likelihood Estimation (FIML) to deal with missing data. However, it is not possible to use FIML with weights. The estimates obtained using FIML are generally very similar to the Maximum Likelihood estimates presented here.

## 3. Results

### 3.1. Correlations and Associations

Table 2 presents the weighted correlations of study variables. The Project Talent correlations are below the main diagonal and those for the NLSY79 are above the main diagonal. The SES-cognitive ability correlation is stronger in the more recent NLSY79 cohort (0.54 compared to 0.31) and the constituent SES variables exhibit higher inter-correlations. Generally, SES has stronger correlations with educational and labor market outcomes in the NLSY79 than Project Talent. The stronger associations of SES with educational and labor market outcomes in the NLSY79 are also evident in the regression analyses reported below.

### 3.2. Grades-at-School

Table 3 presents the estimates from the analyses of grades-at-school. Model 1 shows that SES is only weakly associated with grades-at-school in Project Talent, accounting for only 1% of the variance. In contrast, SES accounts for 12% of the variance in the NLSY79. An obvious explanation for the weaker relationship in Project Talent is the greater unreliability of the measure. However, if the reliability of the NLSY79 measure was the same as the Project Talent measure, the standardized effect for SES (0.35 × 0.82 = 0.20) would still be considerably larger than that for Project Talent (β = 0.13).

Model 2 shows that the standardized coefficient for cognitive ability on grades-at-school in the NLSY79 (β = 0.58) is about 1.7 times that for Project Talent (β = 0.34).

Model 3 shows that cognitive ability is a much more powerful predictor of grades-at-school vis-a-vis SES. The SES coefficients are small or very small (β = 0.02 for Project Talent and β = 0.06 for the NLSY79). The standardized coefficients for cognitive ability are much larger (0.33, 0.52).

The addition of ability dramatically reduced the magnitude of the SES coefficient (comparing model 3 estimates to that in model 1). For Project Talent, the metric coefficient declined from 1.35 (on a 50-point scale) to 0.18. For the NLSY79 the metric coefficient declined from 0.33 on a 5-point scale to 0.06.

In contrast, comparison of models 2 and 3 reveals only marginal declines in the cognitive ability coefficients with the addition of SES. For Project Talent, the cognitive ability coefficient declined from 3.45 to 3.39 and in the NLSY79 from 0.53 to 0.50. Furthermore, the addition of SES did not increase the variance explained in grades-at-school from that in the ability-only models. So, the effects of cognitive ability are robust, whereas SES effects are, to a considerable extent, confounded by cognitive ability.

### 3.3. Educational Attainment

Table 4 presents the estimates for educational attainment. SES has a stronger association with years of education in the NLSY79 when compared to Project Talent. According to model 1, a one-standard deviation increase in SES translates to an increase of 1.27 years of education in the NLSY79 compared to 0.85 years in Project Talent. In contrast, a one-standard deviation increase in cognitive ability is associated with 1.5 years more education in both studies (model 2). Cognitive ability accounts for nearly 40% of the variance in years of education in both Project Talent and the NLSY79.

Model 3 shows that cognitive ability has much stronger effects on educational attainment than SES. For Project Talent, a one-standard deviation in cognitive ability translates to 1.36 years additional education compared to 0.39 years for a corresponding difference in SES. For the NLSY79, the estimates are 1.15 years for cognitive ability and 0.64 years for SES. These differences are also apparent from the standardized coefficients: 0.16 for SES and 0.56 for ability in Project Talent and 0.26 and 0.47 in the NLSY79.

Comparing models 1 and 3, the addition of cognitive ability tripled the variance in educational attainment accounted for in Project Talent and by about 50% in the NLSY79. In contrast, the addition of SES only marginally increased the variance explained compared to the ability only models, from 38% to 40% in Project Talent and from 37 to 41% in the NLSY79. As for grades-at-school, SES effects are confounded by cognitive ability, whereas the effects of cognitive ability decline only decline marginally with the addition of SES.

### 3.4. Occupational Attainment

Table 5 presents the estimates for occupational attainment at age 29. As for education, SES has a stronger association with occupational attainment in the NLSY79 than in Project Talent. According to model 1, a one-standard deviation increase in SES translates to an increase of 5.2 units in occupational attainment in Project Talent and 6.7 units in the NLSY79. Correspondingly, SES accounts for 6% of the variation in occupational attainment in Project Talent and 13% in the NLSY79.

The effects of cognitive ability are larger and similar across studies. A one-standard deviation increase in cognitive ability is associated with a 9.7 unit increase in occupational attainment in Project Talent and an 8.8 unit increase in the NLSY79. The standardized coefficients are almost identical (0.44, 0.46). Cognitive ability accounts for about 20% of the variance in occupational attainment in both Project Talent and the NLSY79.

Model 3 shows that cognitive ability has much stronger effects on occupational attainment vis-a-vis SES. For Project Talent, a one-standard deviation increase in cognitive ability translates to an increase of 8.9 units in occupational attainment. The estimate for the NLSY79 is 7.2 units. This compares to effects of 2.6 and 3.0 units for SES. The standardized coefficient for cognitive ability is more than three times larger than that for SES in Project Talent and more than twice as large in the NLSY79. As for school grades and educational attainment, the SES coefficient, net of cognitive ability, is larger in the more recent NLSY79 cohort (0.16 cf. 0.12).

With the addition of educational attainment in model 4, SES effects become trivial with standardized coefficients of 0.04. By contrast, the standardized coefficients for cognitive ability, net of SES and education are substantial: 0.20 in Project Talent and 0.18 in the NLSY79. So, in both cohorts, cognitive ability increases occupational attainment, net of educational attainment.

### 3.5. Income

Table 6 presents the estimates for IHS transformed (or logged) income at age 29. A one-standard deviation increase in SES is associated with a 7.5% increase in income in Project Talent and a 24% increase in the NLSY79 (model 1). However, SES only weakly accounts for variation in personal income, 2% in Project Talent and 5% in the NLSY79. So, SES is more weakly associated with income at age 29 than with grades-at-school, education, or occupation.

Predictably, the comparable effects of cognitive ability are larger: a one-standard deviation increase in cognitive ability is associated with an 8.2% increase in Project Talent and 34% increase in the NLSY79 (model 2).

Consistent with previous analyses, model 3 shows stronger effects of cognitive ability vis-a-vis SES. In Project Talent, a one-standard deviation increase translates to income increases of 4.7% for SES and 8.4% for cognitive ability. In the NLSY79, cognitive ability has a much larger impact than SES, 29.6% compared to 8.4%. The relatively stronger impact of cognitive ability in the NLSY79 is also evident from comparing the standardized coefficients: 0.08 (SES) and 0.10 (ability) in Project Talent and 0.08 and 0.27 in the NLSY79.

In both studies, SES effects are small (β ≈ 0.05) net of cognitive ability and educational attainment (model 4). In Project Talent, the net effect of cognitive ability is zero, but is significant in the NLSY79: a one-standard deviation increase in cognitive ability is associated with a substantial 24% increase in income, net of educational attainment and SES.

Note that the metric coefficients for educational attainment in model 4 are estimates of the rate-of-return to education for a one-year increase in education, net of SES and ability. The rate-of-return for education is 6.6% in Project Talent and 4.9% in the NLSY79. The regression estimates for the rates of return from other studies are between 4.1% and 10.6% (Ashenfelter and Rouse 2000, pp. 98, 101).

Model 5 adds occupational attainment. In Project Talent there are only trivial effects for both SES and cognitive ability on income, net of education, and occupational attainment. In contrast, in the NLSY79 there is a substantial net effect of cognitive ability. A one-standard deviation increase in cognitive ability is associated with a sizable 20% increase in income, net of SES, education and occupation.

## 4. Discussion

The conclusions from this study could be challenged by questioning the comparability of the samples and their measures. The NLSY79 was never designed to be compared to Project Talent. The sampled populations are different, NLSY79 respondents were older when first contacted, the cognitive items and survey questions are different, and the data were not collected within schools. There was very high attrition for the 11-year Project Talent follow-up. However, few longitudinal surveys are identical. The NLSY79 study differs from its sister 1997 study in the population sampled and often the questions asked, but this does not preclude informative comparisons (Belley and Lochner 2007; Belzil and Hansen 2020; Marks 2022).

For this study, the stronger effects of cognitive ability in the NLSY79 cannot be attributed to NLSY79 respondents being older at the time of initial contact and different cognitive items. If that were true, there would be stronger effects of cognitive ability in the NLSY79 for all outcomes, but for educational attainment and occupational attainment its effects are similar to its effects in Project Talent. 

Measurement error is problematic for comparing the analyses of school grades, which is measured accurately in the NLSY79 but by student reports in Project Talent. Logic suggests that measurement error accounts for the stronger effects of SES and cognitive ability on school grades in the NLSY79. However, the differences are too large to be fully accounted for by measurement error. Similarly, income is measured by more questions in the NLSY79 than in Project Talent. However, the ability-income correlation in Project Talent (0.14) among 29-year-olds is not dramatically lower than Strenze’s (2007, p. 412) estimate of 0.20 for 20- to 78-year-olds. Furthermore, the 6.6% rate-of-return for education estimated in the Project Talent data is very plausible given rate-of-return estimates from other studies and the youth of the sample.

Consistent with the modernization thesis, socioeconomic background is not the dominant influence on educational and occupational attainments. Once cognitive ability is considered, the effects of SES on grades-at-school and income are small (β < 0.10). For educational and occupational attainment, SES effects are larger, but are much smaller than suggested by the raw correlations or analyses that do not include cognitive ability. If socioeconomic background is the dominant influence on educational and socioeconomic attainments, then controlling for cognitive ability should not reduce the size of the SES coefficients. Instead, SES estimates decline by about one-half, sometimes more.

Consistent with the meritocracy thesis, cognitive ability is a much stronger influence on educational and labor market outcomes than SES. This is true for a cohort born as long ago as the 1940s. Furthermore, its effects appear to be increasing. Cognitive ability has a larger impact on school grades and income in the more recent NLSY79 cohort. Cognitive ability influences income, net of education in the more recent NLSY79, but not in the older Project Talent cohort.

It is interesting to speculate when cognitive ability became important for social stratification. Its importance for primary school education was evident soon after IQ tests were developed in the early 1900s. It may be that in educational contexts cognitive ability is always important since educational assessments invariably involve understanding and reasoning. For cognitive ability to influence labor market outcomes, there are two preconditions: an achievement orientated society where education is the primary mechanism for socioeconomic attainments and near-universal educational participation. This implies that there is a sequence; cognitive ability becomes important first for primary school education, then secondary school education and subsequently for labor market outcomes.

For each stratification outcome, SES effects are larger in the more recent cohort. However, this finding does not necessarily mean that family-of-origin economic and cultural differences have become more salient for educational and occupational outcomes. Educational and occupational attainments in adulthood are strongly correlated with cognitive ability measured during childhood or adolescence (Strenze 2007). Marks and O’Connell (2023) showed that SES effects on measures of children’s cognitive ability and school achievement decline by over 50% with the addition of mother’s ability. Greater declines are likely if a measure of father’s ability was available.

Therefore, the SES coefficients reported here may, to a considerable extent, index parents’ abilities rather than economic and cultural resources. This provides an explanation for stronger SES effects in the NLSY79. SES is more closely associated with offspring’s ability in the NLSY79 than Project Talent because cognitive ability was more important for socioeconomic attainments among NLSY79 parents than among Project Talent parents. The relationship between SES and parents’ abilities also explains the phenomenon repeatedly observed here: the addition of cognitive ability substantially reduces SES coefficients. That is because SES effects are confounded by parents’ abilities.

This study repeatedly demonstrated that the addition of SES only marginally reduces the effects of cognitive ability. The effects of cognitive ability are robust because there is a sizable genetic component to cognitive ability (Deary 2012; Plomin and Deary 2015). There are also sizable genetic components to the stratification outcomes analyzed here: grades and student achievement (de Zeeuw et al. 2015; Pokropek and Sikora 2015; Little et al. 2017), educational attainment (Branigan et al. 2013; Pokropek and Sikora 2015; Silventoinen et al. 2020), occupational attainment (Behrman et al. 1977, p. 71; Roos and Nielsen 2019), and income (Hyytinen et al. 2019; Roos and Nielsen 2019). So, both the effects of ability and SES effects incorporate genetics further complicating conclusions on the modernization and meritocratic theses.

### Inconsistent Conclusions on Modernization and the Meritocracy Theses

Why are conclusions on the modernization and meritocracy theses so inconsistent? There is a large literature in economics, education and sociology on ‘persistent inequality’ which is often very critical of modernization theory (Piketty 2000; Gamoran 2001, p. 135; Shavit et al. 2007; Pfeffer 2008). This literature is in stark contrast to the modernization/meritocracy literature (Treiman 1970; Saunders 1995; Sieben and de Graaf 2001; Kingston 2006; Alon and Tienda 2007; Marks 2014; Herrala 2023). There are several reasons for the inconsistencies.

First, the great bulk of studies on persistent inequality do not include cognitive ability, so its effects are incorporated into SES or social class background. Relatedly, cognitive ability is often incorrectly specified as a consequence of, rather than independent of, SES or social class background (Bourne et al. 2018; Betthäuser et al. 2020), so its effects are interpreted as simply mediating the effects of SES (see Gottfredson 2016).

Second, there is evidence of declines in the effects of socioeconomic background for social stratification. According to Hout (2018) father-son correlations for occupational status in the US were 0.42 in 1962, 0.38 in 1973, 0.33 in the 1980s and 0.31 from 1994 to 2016. Breen et al. (2009, 2010) found declines in socioeconomic inequalities in education in several European countries for both men and women. Contrary to the prevailing wisdom, Buscha and Sturgis (2018) found increases in social mobility in Britain between the 1950s and 1980s.

Third, the common assumption that in a fair and just society there would be no association between parents and their offspring’s socioeconomic attainments is untenable (Eckland 1967). According to this assumption, either ability is irrelevant to social stratification or randomly distributed across socioeconomic groups. Similarly, Swift (2004) notes that some of the mechanisms that produce an association between the social position of parents and their children that are unobjectionable and would exist in a just society. Murray (2020, p. 237) pointed out that any measure of parental SES is not only a measure of the child’s environment, but is also measures parents’ abilities and talents, all of which have genetic components. Furthermore, the associations between parents’ education and occupation, and their children’s test scores remains largely intact in purportedly egalitarian environments, such as communist Poland and the Kibbutz (Firkowska et al. 1978; Justman and Gilboa 2012).

Fourth, the parent-child correlations for cognitive ability, student achievement, educational and occupational attainment are not large as generally assumed but only moderate, around 0.3 or 0.4, but sometimes as low as 0.2. These correlations are much smaller than that implied by conventional theories of socioeconomic inequality. Such discrepancies are almost never addressed.

Finally, the correlations between pairs of relatives are well predicted by Fisher’s (1918) polygenetic model in which incorporates genetic heritability and assortative mating. Blau and Duncan (1967, p. 147) note that it is at least curious that the father-son correlation for occupational status in the US is comparable to that for statue for which genetic transmission is strongly indicated. The theoretical correlations derived from Fisher type models for IQ, education and social status are very close to the observed correlations (Jensen 1969, p. 49; Behrman and Taubman 1989; Clark 2023).

The lack of consensus can be attributed to these factors. Hopefully as the process of social stratification is better understood, especially the importance of cognitive ability and genetics, there will be greater consensus.

## Figures and Tables

**Table 1:** SEM Loadings of Subtests in Project Talent.

Subtest	Loadings	
	Present Study	Jencks et al. (1983)
Abstract Reasoning	0.68	0.65
Advanced High School Math (Math III)	0.55	0.66
Arithmetic Computation	0.56	0.57
Arithmetic Reasoning (Math I)	0.78	0.75
Creativity	0.72	0.71
Disguised Words	0.65	-
English Literature	0.79	0.80
Introduction High School Math (Math II)	0.79	0.79
Math Information	0.80	0.83
Mechanical Reasoning	0.63	0.64
Reading Comprehension	0.85	0.86
Visualization in 2 Dimensions	0.46	-
Visualization in 3 Dimensions	0.58	0.55
Vocabulary I	0.85	0.90
Vocabulary II	0.75	

**Table 2:** Correlations and Univariate Statistics of Study Variables.

	SES	Father’s Education	Mother’s Education	Father’s Occupation (SEI)	Mother’s Occupation (SEI)	Family Income	Family Income (HIS)	Cognitive Ability (g)	High School Grades	Years of Education	Occupation Age 29 (SEI)	Income Age 29	Income Age 29 (IHS)
**SES**		0.84	0.80	0.80	0.75	0.60	0.60	0.54	0.35	0.51	0.36	0.26	0.22
**Father’s Education**	0.77		0.61	0.62	0.42	0.39	0.31	0.46	0.27	0.45	0.31	0.21	0.18
**Mother’s Education**	0.74	0.55		0.43	0.51	0.37	0.32	0.45	0.28	0.45	0.30	0.18	0.15
**Father’s Occupation**	0.71	0.29	0.22		0.39	0.41	0.32	0.41	0.25	0.42	0.30	0.20	0.17
**Mother’s Occupation**	0.70	0.30	0.35	0.30		0.32	0.23	0.36	0.24	0.34	0.27	0.17	0.14
**Family Income**	0.58	0.28	0.22	0.22	0.21		0.77	0.37	0.23	0.31	0.24	0.26	0.22
**Family Inc (IHS)**	0.61	0.21	0.18	0.22	0.14	0.85		0.34	0.23	0.25	0.18	0.20	0.18
**Cog. Ability (g)**	0.31	0.29	0.27	0.20	0.09	0.21	0.28		0.58	0.61	0.46	0.35	0.31
**HS Grades**	0.13	0.13	0.11	0.06	0.05	0.06	0.07	0.34		0.57	0.38	0.17	0.16
**Years of Education**	0.35	0.38	0.35	0.20	0.18	0.23	0.24	0.61	0.39		0.57	0.31	0.26
**Occupation**	0.24	0.26	0.21	0.15	0.10	0.15	0.16	0.44	0.26	0.62		0.36	0.32
**Income age 29**	0.12	0.10	0.10	0.07	0.06	0.14	0.11	0.14	0.03	0.26	0.20		0.79
**Income (IHS)**	0.11	0.09	0.09	0.07	0.05	0.13	0.10	0.14	0.01	0.23	0.20	0.84	0.12
**Project Talent**													
**N**	95911	79893	83157	95642	44367	58286	58286	89596	91382	65458	64986	61478	61478
**Mean**	0.10	10.68	10.84	48.26	39.72	7516	9.27	0.00	25.27	13.82	49.38	10357	9.78
**Std**	0.97	3.94	3.6	24.35	2.73	5299	0.94	0.98	9.94	2.29	20.57	5381	0.562
**NLSY79**													
**N**	9977	8415	9280	6898	5460	6132	6132	9298	7776	9999	8416	8142	8142
**Mean**	0.29	11.93	11.66	40.52	37.31	21207	10.41	0.27	2.43	13.14	46.41	42056	13.20
**Std**	0.97	3.56	2.69	24.88	22.12	13281.7	0.88	0.97	0.86	2.41	18.23	30695	2.44

Note: Project Talent correlations below diagonal, NLSY79 correlations above diagonal. Occupation = Duncan Socioeconomic Index of Occupation. SEI = Socioeconomic Index for Occupations. IHS = Inverse Hyperbolic Sine transformation. Zero incomes excluded. Weights for Age 29.

**Table 3:** School Grades on Socioeconomic Background and Cognitive Ability.

	*Project Talent*						*NLSY79*					
	Model 1		Model 2		Model 3		Model 1		Model 2		Model 3	
	*Est*	*Std*	*Est*	*Std*	*Est*	*Std*	*Est*	*Std*	*Est*	*Std*	*Est*	*Std*
Intercept	25.19	-	25.30	-	25.20	-	2.31	-	2.24	-	2.23	-
SES	1.35	*0.13*			0.18	*0.02*	0.33	*0.35*			0.06	*0.06*
Ability (*g*)			3.45	*0.34*	3.39	*0.33*			0.53	*0.58*	0.50	*0.52*
Number of Observations	91,370		85,700		85,689		7756		7321		7303	
R Square	0.01		0.12		0.12		0.12		0.34		0.34	

**Table 4:** Educational Attainment on Socioeconomic Background and Cognitive Ability.

	*Project Talent*						*NLSY79*					
	Model 1		Model 2		Model 3		Model 1		Model 2		Model 3	
	*Est*	*Std*	*Est*	*Std*	*Est*	*Std*	*Est*	*Std*	*Est*	*Std*	*Est*	*Std*
Intercept	13.83	-	13.79	-	13.81	-	12.78	-	12.81	-	12.71	-
SES	0.853	*0.35*			0.39	*0.16*	1.27	*0.51*			0.64	*0.26*
Ability (*g*)			1.49	*0.61*	1.36	*0.56*			1.49	*0.61*	1.15	*0.47*
Number of Observations	71,303		66,697		66,673		9946		9271		9234	
R Square	0.12		0.38		0.40		0.26		0.37		0.41	

**Table 5:** Occupational Attainment at age 29 on Socioeconomic Background, Cognitive Ability and Educational Attainment.

	Project Talent								NLSY79							
	Model 1		Model 2		Model 3		Model 4		Model 1		Model 2		Model 3		Model 4	
	*Est*	*Std*	*Est*	*Std*	*Est*	*Std*	*Est*	*Std*	*Est*	*Std*	*Est*	*Std*	*Est*	*Std*	*Est*	*Std*
Intercept	49.39	-	48.75	-	48.77	-	−11.27	-	44.13	-	43.78	-	43.23	-	−0.127	-
SES	5.22	*0.24*			2.62	*0.12*	0.86	*0.04*	6.71	*0.36*			2.97	*0.16*	0.72	*0.04*
Ability (*g*)			9.70	*0.44*	8.93	*0.41*	4.43	*0.20*			8.80	*0.46*	7.23	*0.38*	3.36	*0.18*
Educational attainment							4.31	*0.48*							3.40	*0.45*
Number of Observations	64,534		60,284		60,268		45,671		8378		7836		7811		7791	
R Square	0.06		*0.20*		*0.21*		0.41		0.13		*0.22*		*0.23*		0.35	

**Table 6:** Income at age 29 on Socioeconomic Background, Cognitive Ability, and Educational and Occupation Attainment.

	*Model 1*		*Model 2*		*Model 3*		*Model 4*		*Model 5*	
	Est	Std	Est	Std	Est	Std	Est	Std	Est	Std
**Project Talent**										
Intercept	9.79	-	9.78	-	9.78	-	8.97	-	8.87	**-**
SES	0.075	*0.13*			0.047	*0.08*	0.025	*0.04*	0.014	*0.04*
Ability (*g*)			0.082	*0.14*	0.062	*0.10*	0.001 ^ns^	*0.00*	0.004 ^ns^	*−0.02*
Educational attainment							0.066	*0.24*	0.045	*0.18*
Occupational Attainment									0.004	*0.13*
Number of Observations	58,856		57,052		55,135		41,800		41,575	
R Square	0.02		0.02		0.02		0.07		0.08	
**NLSY79**										
Intercept	10.91	-	10.87	-	10.86	-	10.24	-	10.32	-
SES	0.241	*0.22*			0.084	*0.08*	0.051	*0.05*	0.038	*0.04*
Ability (*g*)			0.341	*0.31*	0.296	*0.27*	0.241	*0.22*	0.200	*0.19*
Educational attainment							0.049	*0.11*	0.004 ^ns^	*0.01*
Occupational Attainment									0.012	*0.22*
Number of Observations	8111		7592		7572		7554		7318	
R Square	0.05		0.10		0.10		0.11		0.14	

Note: ns = Not statistically significant.

## Data Availability

The Project Talent data may be obtained from the American Institutes for Research https://www.air.org/resource/spotlight/project-talent-data-available-researchers. The NLSY79 data is available from the U.S. Bureau of Labor Statistics https://www.nlsinfo.org/content/cohorts/nlsy79.

## Notes

1 In this paper the concepts intelligence and cognitive ability are synonymous.

2 SEI scores narrowly-defined census occupational groups by the incomes and educations of their incumbents.

3 The question wording was “*What were your total personal earnings (before deductions) from all your jobs for the year between 1 September 1973 and 31 August 1974 (Please include all wages, salaries, commissions, tips, as well as net income from farming or other businesses. Do not include income other than earnings such as social security, public assistance, interest, welfare, or veterans’ income payments). If you don’t know exactly, please make an estimate.*”

4 The Inverse Hyperbolic Sine transformation is calculated as follows: IHS(x) = log(x + sqrt(x^2^ + 1)).

5 The project talent SES composite was constructed from responses to questions on home value, family income, number of books in the house, number of appliances, number of electronics, availability of a private room for the child, father’s job status, father’s education, and mother’s education (Wise et al. 1979, pp. 37–38; Damian et al. 2015).
